# 'Boiled egg' in the peritoneal cavity-a giant peritoneal loose body in a 64-year-old man: a case report

**DOI:** 10.1186/1752-1947-5-297

**Published:** 2011-07-07

**Authors:** Ajit Sewkani, Aruna Jain, KK Maudar, Subodh Varshney

**Affiliations:** 1 Department of Surgical Gastroenterology & Clinical Nutrition, Bhopal Memorial Hospital and Research Center, Bhopal (MP) 462038, India; 2 Department of Pathology, Bhopal Memorial Hospital and Research Center, Bhopal (MP) 462038, India

## Abstract

**Introduction:**

Peritoneal loose bodies, or peritoneal mice, are rare asymptomatic lesions that are usually found as an incidental finding during abdominal surgery or autopsy. Giant loose bodies, measuring more than 5 cm, are rare and only a few cases are reported in the literature. These bodies are usually infarcted appendices epiploicae, which become detached and appear as a peritoneal loose body in the abdominal cavity. They may re-attach themselves to a surface, such as the lower aspect of the spleen or omentum, in which case they can be called a "parasitized peritoneal body", as in our case.

**Case Presentation:**

We report a case of a giant loose peritoneal body measuring 7 × 5 cm found incidentally in a 64-year-old Indian man who presented with acute intestinal obstruction. We present the current hypothesis and our opinion on the genesis of such large bodies and discuss the problems in diagnosis.

**Conclusion:**

Peritoneal loose bodies are common but giant peritoneal loose bodies are very rare. These giant bodies usually do not require any treatment until they become complicated. Present diagnosis modalities have limitations in the diagnosis of mobile lesions in the abdominal cavity, so care must be taken to avoid unnecessary laparotomies in uncomplicated cases.

## Introduction

Peritoneal loose bodies are rare and found incidentally at laparotomy. In most cases they are small in size (usually less than 1 cm). Giant loose bodies (more than 5 cm) are very rare and only a few cases have been reported in the literature [1-10]. Its exact pathogenesis is not known but the most common origin of these bodies are appendices epiploicae (by the sequential process of torsion, infarction, saponification and calcification) [1,2].

These loose bodies are usually incidental findings that do not require any specific treatment until they become complicated [3-6]. Generally, computed tomography (CT) and magnetic resonance imaging (MRI) are useful for diagnosis of these lesions; however present literature shows the limitation in the diagnosis of movable masses by CT and MRI [2,7]. We report a case of a giant loose peritoneal body with special reference to the genesis of such large bodies and also discuss the problems in diagnosis.

## Case Report

**Figure 1:**
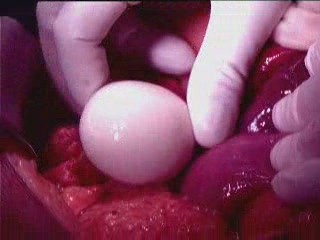
**Macrograph showing location of the 'boiled egg' (Giant loose peritoneal body) in the abdomen which was something of a surprise to the operating surgeon during exploration and adhesiolysis for acute intestinal obstruction**.

**Figure 2:**
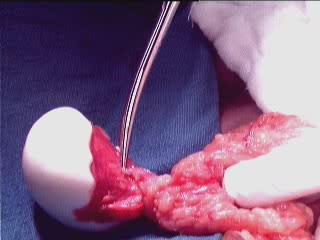
**Macrograph showing giant peritoneal loose body parasitized to omentum**. The macrograph clearly shows the giant loose body attached to omentum and a separate twig from omentum supplying to loose peritoneal body.

A 64-year-old Indian man was referred with complaints of abdominal pain, vomiting and not passing flatus or feces for four days. Our patient's general condition was poor; he was febrile, with a pulse rate of 124/minute and blood pressure 90 mm/Hg. X-rays of his abdomen showed multiple air fluid levels suggestive of acute intestinal obstruction. With the provisional diagnosis of acute abdomen (acute intestinal obstruction) our patient was resuscitated and sent for an urgent laparotomy. On exploration, our patient had severely dilated small gut loops with the terminal ileal loop twisted around the omental band and adherent to his left pelvic wall. On releasing the omental band, the ileal loop was dissected free from his left pelvic wall. Once the loops had been released, we found a large, white, oval shaped, extra-luminal body in the region of his sigmoid colon (Figure 1). The body was soft to firm in consistency (resembling a boiled hen's egg) and attached (parasitized) to the omentum (Figure 2). In addition, part of the appendices epiploicae, attached to his sigmoid colon, were calcified with constricted stalks. The peritoneal loose body was largely parasitized to the omentum with a separate feeding vessel supplying it from the omentum.

**Figure 3:**
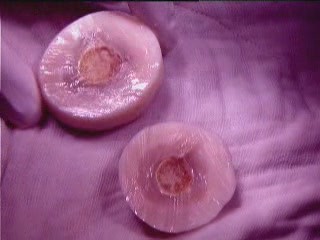
**Macrograph of giant loose body (cut surface) showing peripheral white & central yellow portions resembling a boiled egg**.

The body was an oval shaped mass, measuring 7 cm in length and 5 cm in width, and weighed 74 g. On the cut surface, it had classic appearance like a boiled egg, with a distinct white peripheral part and yellow central part (Figure 3). The white part was smooth and soft in consistency while the central yellow part was slightly firm in the periphery and hard (calcified) at the central point. The surfaces were smooth and shiny. On histological examination, it consisted of laminated strands of a fibrinoid substance with a large amount of proteinaceous material in the peripheral white part (boiled albumin with a high collagen deposition) and saponified fat with calcification in the central yellow part.

Our patient did well post-operatively; he resumed his oral diet on the third post-operative day. He was discharged from the hospital five days after the operation.

## Discussion

Giant loose bodies (peritoneal mice) are very rare and only a few cases have been reported in literature [1-10]. The exact pathogenesis of loose bodies has not been fully demonstrated, however the current hypothesis as mention previously by many authors [6,8-10] suggests that it is a sequential process which starts with torsion of an epiploica, followed by ischemia, saponification and calcification. The pedicle atrophies and finally it detaches from the colon surface to become a loose body.

We believe that once an appendix epiploica gets saponified and calcified the exudative serum fluid (rich in protein) accumulates around it and, because of increased temperature in the peritoneal cavity, it gives the appearance of a boiled egg. With time, the size of the peritoneal body increases because of a gradual deposition of body serum at the periphery. Sometimes the free peritoneal body attaches to the omentum and receives a blood supply from it (a parasitized peritoneal body), as in our case.

Our histological findings suggest that saponified and calcified appendices epiploicae form the yellow central part and gradual deposition of peritoneal serum around it form the outer white layer, hence giving the appearance of a boiled hen's egg.

Pre-operative diagnosis of these lesions is difficult, because most of the time these lesions are asymptomatic and found during routine exploration of the abdomen for some other pathology.

The most common form of presentation in symptomatic patients is causing intestinal obstruction, as in this case. If a patient presents with features of intestinal obstruction and X-ray films shows a calcified lesion in the abdomen, which moves with a change in position of the patient, there should be a high index of suspicion for diagnosis of a giant loose peritoneal body. Additional tests which can be done to diagnose peritoneal mice are CT and MRI scans, which can be useful for differentiating these from other lesions. However, it is very difficult to differentiate between these loose bodies and other abdominal benign lesions with calcification, like granuloma or tuberculosis.

In our case, because the patient presented with acute intestinal obstruction and an X-ray of his abdomen only showed multiple air-fluid levels and no calcified lesions, our patient was directly taken up for an urgent laparotomy without waiting for CT or MRI scans.

## Conclusion

Peritoneal loose bodies are rare and, in most of the cases, small in size. However, giant loose bodies are very rare and only a few cases have been reported in the literature. The current hypothesis on their development is uncertain.

Pre-operative diagnosis of these lesions is difficult and a high index of suspicion should be kept in any symptomatic patient with a mobile lesion in the abdomen or a calcified lesion in the pelvis on X-ray.

No specific treatment is required in asymptomatic patients, however if these entities become associated with complications like intestinal obstruction, or if there is an abdominal mass of obscure origin, or when diagnosis is in doubt, then exploration is required.

## Consent

Written informed consent for publication could not be obtained despite all reasonable attempts. Every effort has been made to protect the identity of our patient and there is no reason to believe that our patient would object to publication.

## Competing interests

The authors declare that they have no competing interests.

## Authors' contributions

AS assisted in the operation, contributed to manuscript conception, research, acquisition of data, drafting and writing of the manuscript as well as pre-operative and post-operative management of the patient. AJ carried out the histopathological evaluation and critically review of the manuscript. KKM carried out the critical review and revision of the manuscript. SV carried out the operation and contributed to the critical review of the manuscript. All authors read and approved the final manuscript.
